# Accelerated immune ageing is associated with COVID-19 disease severity

**DOI:** 10.1186/s12979-023-00406-z

**Published:** 2024-01-11

**Authors:** Janet M. Lord, Tonny Veenith, Jack Sullivan, Archana Sharma-Oates, Alex G. Richter, Neil J. Greening, Hamish J. C. McAuley, Rachael A. Evans, Paul Moss, Shona C. Moore, Lance Turtle, Nandan Gautam, Ahmed Gilani, Manan Bajaj, Louise V. Wain, Christopher Brightling, Betty Raman, Michael Marks, Amisha Singapuri, Omer Elneima, Peter J. M. Openshaw, Niharika A. Duggal, on behalf of the PHOSP-COVID Study collaborative group, K. Abel, H. Adamali, D. Adeloye, O. Adeyemi, R. Adrego, L. A. AguilarJimenez, S. Ahmad, N Ahmad Haider, R. Ahmed, N. Ahwireng, M. Ainsworth, B. Al-Sheklly, A. Alamoudi, M. Ali, M. Aljaroof, A. M. All, L. Allan, R. J. Allen, L. Allerton, L. Allsop, P. Almeida, D. Altmann, M Alvarez Corral, S. Amoils, D. Anderson, C. Antoniades, G. Arbane, A. Arias, C. Armour, L. Armstrong, N. Armstrong, D. Arnold, H. Arnold, A. Ashish, A. Ashworth, M. Ashworth, S. Aslani, H. Assefa-Kebede, C. Atkin, P. Atkin, R. Aul, H. Aung, L. Austin, C. Avram, A. Ayoub, M. Babores, R. Baggott, J. Bagshaw, D. Baguley, L. Bailey, J. K. Baillie, S. Bain, M. Bakali, M. Bakau, E. Baldry, D. Baldwin, M. Baldwin, C. Ballard, A. Banerjee, B. Bang, R. E. Barker, L. Barman, S. Barratt, F. Barrett, D. Basire, N. Basu, M. Bates, A. Bates, R. Batterham, H. Baxendale, H. Bayes, M. Beadsworth, P. Beckett, M. Beggs, M. Begum, P. Beirne, D. Bell, R. Bell, K. Bennett, E. Beranova, A. Bermperi, A. Berridge, C. Berry, S. Betts, E. Bevan, K. Bhui, M. Bingham, K. Birchall, L. Bishop, K. Bisnauthsing, J. Blaikely, A. Bloss, A. Bolger, C. E. Bolton, J. Bonnington, A. Botkai, C. Bourne, M. Bourne, K. Bramham, L. Brear, G. Breen, J. Breeze, A. Briggs, E. Bright, S. Brill, K. Brindle, L. Broad, A. Broadley, C. Brookes, M. Broome, A. Brown, A. Brown, J. Brown, J. Brown, J. S. Brown, M. Brown, M. Brown, V. Brown, T. Brugha, N. Brunskill, M. Buch, P. Buckley, A. Bularga, E. Bullmore, L. Burden, T. Burdett, D. Burn, G. Burns, A. Burns, J. Busby, R. Butcher, A. Butt, S. Byrne, P. Cairns, P. C. Calder, E. Calvelo, H. Carborn, B. Card, C. Carr, L. Carr, G. Carson, P. Carter, A. Casey, M. Cassar, J. Cavanagh, M. Chablani, T. Chalder, J. D. Chalmers, R. C. CHambers, F. Chan, K. M. Channon, K. Chapman, A. Charalambou, N. Chaudhuri, A. Checkley, J. Chen, Y. Cheng, L. Chetham, C. Childs, E. R. Chilvers, H. Chinoy, A. Chiribiri, K. Chong-James, G. Choudhury, N. Choudhury, P. Chowienczyk, C. Christie, M. Chrystal, D. Clark, C. Clark, J. Clarke, S. Clohisey, G. Coakley, Z. Coburn, S. Coetzee, J. Cole, C. Coleman, F. Conneh, D. Connell, B. Connolly, L. Connor, A. Cook, B. Cooper, J. Cooper, S. Cooper, D. Copeland, T. Cosier, M. Coulding, C. Coupland, E. Cox, T. Craig, P. Crisp, D. Cristiano, M. G. Crooks, A. Cross, I. Cruz, P. Cullinan, D. Cuthbertson, L. Daines, M. Dalton, P. Daly, A. Daniels, P. Dark, J. Dasgin, A. David, C. David, E. Davies, F. Davies, G. Davies, G. A. Davies, K. Davies, M. J. Davies, J. Dawson, E. Daynes, A. De Soyza, B. Deakin, A. Deans, C. Deas, J. Deery, S. Defres, A. Dell, K. Dempsey, E. Denneny, J. Dennis, A. Dewar, R. Dharmagunawardena, N. Diar-Bakerly, C. Dickens, A. Dipper, S. Diver, S. N. Diwanji, M. Dixon, R. Djukanovic, H. Dobson, S. L. Dobson, A. B. Docherty, A. Donaldson, T. Dong, N. Dormand, A. Dougherty, R. Dowling, S. Drain, K. Draxlbauer, K. Drury, P. Dulawan, A. Dunleavy, S. Dunn, C. Dupont, J. Earley, N. Easom, C. Echevarria, S. Edwards, C. Edwardson, H. El-Taweel, A. Elliott, K. Elliott, Y. Ellis, A. Elmer, D. Evans, H. Evans, J. Evans, R. Evans, R. I. Evans, T. Evans, C. Evenden, L. Evison, L. Fabbri, S. Fairbairn, A. Fairman, K. Fallon, D. Faluyi, C. Favager, T. Fayzan, J. Featherstone, T. Felton, J. Finch, S. Finney, J. Finnigan, L. Finnigan, H. Fisher, S. Fletcher, R. Flockton, M. Flynn, H. Foot, D. Foote, A. Ford, D. Forton, E. Fraile, C. Francis, R. Francis, S. Francis, A. Frankel, E. Fraser, R. Free, N. French, X. Fu, J. Fuld, J. Furniss, L. Garner, J. R. Geddes, J. George, P. George, M. Gibbons, M. Gill, L. Gilmour, F. Gleeson, J. Glossop, S. Glover, N. Goodman, C. Goodwin, B. Gooptu, H. Gordon, T. Gorsuch, M. Greatorex, P. L. Greenhaff, W. Greenhalf, A. Greenhalgh, J. Greenwood, H. Gregory, R. Gregory, D. Grieve, D. Griffin, L. Griffiths, A.-M. Guerdette, B Guillen Guio, M. Gummadi, A. Gupta, S. Gurram, E. Guthrie, Z. Guy, H. Henson, K. Hadley, A. Haggar, K. Hainey, B. Hairsine, P. Haldar, I. Hall, L. Hall, M. Halling-Brown, R. Hamil, A. Hancock, K. Hancock, N. A. Hanley, S. Haq, H. E. Hardwick, E. Hardy, T. Hardy, B. Hargadon, K. Harrington, E. Harris, V. C. Harris, E. M. Harrison, P. Harrison, N. Hart, A. Harvey, M. Harvey, M. Harvie, L. Haslam, M. Havinden-Williams, J. Hawkes, N. Hawkings, J. Haworth, A. Hayday, M. Haynes, J. Hazeldine, T. Hazelton, L. G. Heaney, C. Heeley, J. L. Heeney, M. Heightman, S. Heller, M. Henderson, L. Hesselden, M. Hewitt, V. Highett, T. Hillman, T. Hiwot, L. P. Ho, A. Hoare, M. Hoare, J. Hockridge, P. Hogarth, A. Holbourn, S. Holden, L. Holdsworth, D. Holgate, M. Holland, L. Holloway, K. Holmes, M. Holmes, B. Holroyd-Hind, L. Holt, A. Hormis, A. Horsley, A. Hosseini, M. Hotopf, L. Houchen-Wolloff, K. Howard, L. S. Howard, A. Howell, E. Hufton, A. D. Hughes, J. Hughes, R. Hughes, A. Humphries, N. Huneke, E. Hurditch, J. Hurst, M. Husain, T. Hussell, J. Hutchinson, W. Ibrahim, F. Ilyas, J. Ingham, L. Ingram, D. Ionita, K. Isaacs, K. Ismail, T. Jackson, J. Jacob, W. Y. James, W. Jang, C. Jarman, I. Jarrold, H. Jarvis, R. Jastrub, B. Jayaraman, R. G. Jenkins, P. Jezzard, K. Jiwa, C. Johnson, S. Johnson, D. Johnston, C. J. Jolley, D. Jones, G. Jones, H. Jones, H. Jones, I. Jones, L. Jones, M. G. Jones, S. Jones, S. Jose, T. Kabir, G. Kaltsakas, V. Kamwa, N. Kanellakis, S. Kaprowska, Z. Kausar, N. Keenan, S. Kelly, G. Kemp, S. Kerr, H. Kerslake, A. L. Key, F. Khan, K. Khunti, S. Kilroy, B. King, C. King, L. Kingham, J. Kirk, P. Kitterick, P. Klenerman, L. Knibbs, S. Knight, A. Knighton, O. Kon, S. Kon, S. S. Kon, S. Koprowska, A. Korszun, I. Koychev, C. Kurasz, P. Kurupati, C. Laing, H. Lamlum, G. Landers, C. Langenberg, D. Lasserson, L. Lavelle-Langham, A. Lawrie, C. Lawson, C. Lawson, A. Layton, A. Lea, O. C. Leavy, D. Lee, J.-H. Lee, E. Lee, K. Leitch, R. Lenagh, D. Lewis, J. Lewis, K. E. Lewis, V. Lewis, N. Lewis-Burke, X. Li, T. Light, L. Lightstone, W. Lilaonitkul, L. Lim, S. Linford, A. Lingford-Hughes, M. Lipman, K. Liyanage, A. Lloyd, S. Logan, D. Lomas, N. I. Lone, R. Loosley, H. Lota, W. Lovegrove, A. Lucey, E. Lukaschuk, A. Lye, C. Lynch, S. MacDonald, G. MacGowan, I. Macharia, J. Mackie, L. Macliver, S. Madathil, G. Madzamba, N. Magee, M. M. Magtoto, N. Mairs, N. Majeed, E. Major, F. Malein, M. Malim, G. Mallison, W. D.-C. Man, S. Mandal, K. Mangion, C. Manisty, R. Manley, K. March, S. Marciniak, P. Marino, M. Mariveles, E. Marouzet, S. Marsh, B. Marshall, M. Marshall, J. Martin, A. Martineau, L. M. Martinez, N. Maskell, D. Matila, W. Matimba-Mupaya, L. Matthews, A. Mbuyisa, S. McAdoo, H. McAllister-Williams, A. McArdle, P. McArdle, D. McAulay, G. P. McCann, J. McCormick, W. McCormick, P. McCourt, L. Mcgarvey, C. McGhee, K. Mcgee, J. McGinness, K. McGlynn, A. McGovern, H. McGuinness, I. B. McInnes, J. McIntosh, E. McIvor, K. McIvor, L. McLeavey, A. McMahon, M. J. McMahon, L. McMorrow, T. Mcnally, M. McNarry, J. McNeill, A. McQueen, H. McShane, C. Mears, C. Megson, S. Megson, P. Mehta, J. Meiring, L. Melling, M. Mencias, D. Menzies, M Merida Morillas, A. Michael, C. Miller, L. Milligan, C. Mills, G. Mills, N. L. Mills, L. Milner, S. Misra, J. Mitchell, A. Mohamed, N. Mohamed, S. Mohammed, P. L. Molyneaux, W. Monteiro, S. Moriera, A. Morley, L. Morrison, R. Morriss, A. Morrow, A. J. Moss, K. Motohashi, N. Msimanga, E. Mukaetova-Ladinska, U. Munawar, J. Murira, U. Nanda, H. Nassa, M. Nasseri, A. Neal, R. Needham, P. Neill, S. Neubauer, D. E. Newby, H. Newell, T. Newman, J. Newman, A. Newton-Cox, T. Nicholson, D. Nicoll, A. Nikolaidis, C. M. Nolan, M. J. Noonan, C. Norman, P. Novotny, J. Nunag, L. Nwafor, U. Nwanguma, J. Nyaboko, C. O’Brien, K. O’Donnell, D. O’Regan, L. O’Brien, N. Odell, G. Ogg, O. Olaosebikan, C. Oliver, Z. Omar, L. Orriss-Dib, L. Osborne, R. Osbourne, M. Ostermann, C. Overton, J. Owen, J. Oxton, J. Pack, E. Pacpaco, S. Paddick, S. Painter, A. Pakzad, S. Palmer, P. Papineni, K. Paques, K. Paradowski, M. Pareek, D. Parekh, H. Parfrey, C. Pariante, S. Parker, J. Parmar, S. Patale, B. Patel, M. Patel, S. Patel, D. Pattenadk, M. Pavlides, S. Payne, L. Pearce, J. E. Pearl, D. Peckham, J. Pendlebury, Y. Peng, C. Pennington, I. Peralta, E. Perkins, Z. Peterkin, T. Peto, N. Petousi, J. Petrie, P. Pfeffer, J. Phipps, J. Pimm, K Piper Hanley, R. Pius, H. Plant, S. Plein, T. Plekhanova, M. Plowright, K. Poinasamy, O. Polgar, L. Poll, J. C. Porter, J. Porter, S. Portukhay, N. Powell, A. Prabhu, J. Pratt, A. Price, C. Price, C. Price, D. Price, L. Price, L. Price, A. Prickett, J. Propescu, S. Prosper, S. Pugmire, S. Quaid, J. Quigley, J. Quint, H. Qureshi, I. N. Qureshi, K. Radhakrishnan, N. M. Rahman, M. Ralser, A. Ramos, H. Ramos, J. Rangeley, B. Rangelov, L. Ratcliffe, P. Ravencroft, A. Reddington, R. Reddy, A. Reddy, H. Redfearn, D. Redwood, A. Reed, M. Rees, T. Rees, K. Regan, W. Reynolds, C. Ribeiro, A. Richards, E. Richardson, M. Richardson, P. Rivera-Ortega, K. Roberts, E. Robertson, E. Robinson, L. Robinson, L. Roche, C. Roddis, J. Rodger, A. Ross, G. Ross, J. Rossdale, A. Rostron, A. Rowe, A. Rowland, J. Rowland, M. J. Rowland, S. L. Rowland-Jones, K. Roy, M. Roy, I. Rudan, R. Russell, E. Russell, G. Saalmink, R. Sabit, E. K. Sage, T. Samakomva, N. Samani, C. Sampson, K. Samuel, R. Samuel, A. Sanderson, E. Sapey, D. Saralaya, J. Sargent, C. Sarginson, T. Sass, N. Sattar, K. Saunders, R. M. Saunders, P. Saunders, L. C. Saunders, H. Savill, W. Saxon, A. Sayer, J. Schronce, W. Schwaeble, J. T. Scott, K. Scott, N. Selby, M. G. Semple, M. Sereno, T. A. Sewell, A. Shah, K. Shah, P. Shah, M. Shankar-Hari, M. Sharma, C. Sharpe, M. Sharpe, S. Shashaa, A. Shaw, K. Shaw, V. Shaw, A. Sheikh, S. Shelton, L. Shenton, K. Shevket, A. Shikotra, J. Short, S. Siddique, S. Siddiqui, J. Sidebottom, L. Sigfrid, G. Simons, J. Simpson, N. Simpson, C. Singh, S. Singh, S. J. Singh, D. Sissons, J. Skeemer, K. Slack, A. Smith, D. Smith, S. Smith, J. Smith, L. Smith, M. Soares, T. S. Solano, R. Solly, A. R. Solstice, T. Soulsby, D. Southern, D. Sowter, M. Spears, L. G. Spencer, F. Speranza, L. Stadon, S. Stanel, N. Steele, M. Steiner, D. Stensel, G. Stephens, L. Stephenson, M. Stern, I. Stewart, R. Stimpson, S. Stockdale, J. Stockley, W. Stoker, R. Stone, W. Storrar, A. Storrie, K. Storton, E. Stringer, S. Strong-Sheldrake, N. Stroud, C. Subbe, C. L. Sudlow, Z. Suleiman, C. Summers, C. Summersgill, D. Sutherland, D. L. Sykes, R. Sykes, N. Talbot, A. L. Tan, L. Tarusan, V. Tavoukjian, A. Taylor, C. Taylor, J. Taylor, A. Te, H. Tedd, C. J. Tee, J. Teixeira, H. Tench, S. Terry, S. Thackray-Nocera, F. Thaivalappil, B. Thamu, D. Thickett, C. Thomas, D. C. Thomas, S. Thomas, A. K. Thomas, T. Thomas-Woods, T. Thompson, A. A. R. Thompson, T. Thornton, M. Thorpe, R. S. Thwaites, J. Tilley, N. Tinker, G. F. Tiongson, M. Tobin, J. Tomlinson, C. Tong, M. Toshner, R. Touyz, K. A. Tripp, E. Tunnicliffe, A. Turnbull, E. Turner, S. Turner, V. Turner, K. Turner, S. Turney, H. Turton, J. Ugoji, R. Ugwuoke, R. Upthegrove, J. Valabhji, M. Ventura, J. Vere, C. Vickers, B. Vinson, E. Wade, P. Wade, T. Wainwright, L. O. Wajero, S. Walder, S. Walker, S. Walker, E. Wall, T. Wallis, S. Walmsley, J. A. Walsh, S. Walsh, L. Warburton, T. J. C. Ward, K. Warwick, H. Wassall, S. Waterson, E. Watson, L. Watson, J. Watson, J Weir McCall, C. Welch, H. Welch, B. Welsh, S. Wessely, S. West, H. Weston, H. Wheeler, S. White, V. Whitehead, J. Whitney, S. Whittaker, B. Whittam, V. Whitworth, A. Wight, J. Wild, M. Wilkins, D. Wilkinson, B. Williams, N. Williams, N. Williams, J. Williams, S. A. Williams-Howard, M. Willicombe, G. Willis, J. Willoughby, A. Wilson, D. Wilson, I. Wilson, N. Window, M. Witham, R. Wolf-Roberts, C. Wood, F. Woodhead, J. Woods, D. G. Wootton, J. Wormleighton, J. Worsley, D. Wraith, C. Wrey Brown, C. Wright, L. Wright, S. Wright, J. Wyles, I. Wynter, M. Xu, N. Yasmin, S. Yasmin, T. Yates, K. P. Yip, B. Young, S. Young, A. Young, A. J. Yousuf, A. Zawia, L. Zeidan, B. Zhao, B. Zheng, O. Zongo, ISARIC4C investigators

**Affiliations:** 1 MRC-Versus Arthritis Centre for Musculoskeletal Ageing Research, Office 6, University of Birmingham Research Labs, Institute of Inflammation and Ageing, Queen Elizabeth Hospital, Birmingham, UK; 2 NIHR Birmingham Biomedical Research Centre, University Hospital Birmingham and University of Birmingham, Birmingham, UK; 3 NIHR Surgical Reconstruction and Microbiology Research Centre, University Hospital Birmingham, Birmingham, UK; 4 School of Biosciences, University of Birmingham, Birmingham, UK; 5 Institute of Immunology and Immunotherapy, University of Birmingham, Birmingham, UK; 6 Institute for Lung Health, NIHR Leicester Biomedical Research Centre, Glenfield Hospital, University of Leicester, Leicester, UK; 7 NIHR Health Protection Research Unit in Emerging and Zoonotic Infections, Institute of Infection, Veterinary and Ecological Sciences, University of Liverpool, Liverpool, UK; 8 Queen Elizabeth Hospital, University Hospital Birmingham NHS Foundation Trust, Birmingham, UK; 9 Department of Population Health Sciences, University of Leicester, Leicester, UK; 10 Radcliffe Department of Medicine, University of Oxford, Oxford, UK; 11 London School of Hygiene and Tropical Medicine, University of London, London, UK; 12 National Heart and Lung Institute, Imperial College London, London, UK

## Abstract

**Background:**

The striking increase in COVID-19 severity in older adults provides a clear example of immunesenescence, the age-related remodelling of the immune system. To better characterise the association between convalescent immunesenescence and acute disease severity, we determined the immune phenotype of COVID-19 survivors and non-infected controls.

**Results:**

We performed detailed immune phenotyping of peripheral blood mononuclear cells isolated from 103 COVID-19 survivors 3–5 months post recovery who were classified as having had severe (*n* = 56; age 53.12 ± 11.30 years), moderate (*n* = 32; age 52.28 ± 11.43 years) or mild (*n* = 15; age 49.67 ± 7.30 years) disease and compared with age and sex-matched healthy adults (*n* = 59; age 50.49 ± 10.68 years). We assessed a broad range of immune cell phenotypes to generate a composite score, IMM-AGE, to determine the degree of immune senescence. We found increased immunesenescence features in severe COVID-19 survivors compared to controls including: a reduced frequency and number of naïve CD4 and CD8 T cells (*p* < 0.0001); increased frequency of EMRA CD4 (*p* < 0.003) and CD8 T cells (*p* < 0.001); a higher frequency (*p* < 0.0001) and absolute numbers (*p* < 0.001) of CD28^−ve^ CD57^+ve^ senescent CD4 and CD8 T cells; higher frequency (*p* < 0.003) and absolute numbers (*p* < 0.02) of PD-1 expressing exhausted CD8 T cells; a two-fold increase in Th17 polarisation (*p* < 0.0001); higher frequency of memory B cells (*p* < 0.001) and increased frequency (*p* < 0.0001) and numbers (*p* < 0.001) of CD57^+ve^ senescent NK cells. As a result, the IMM-AGE score was significantly higher in severe COVID-19 survivors than in controls (*p* < 0.001). Few differences were seen for those with moderate disease and none for mild disease. Regression analysis revealed the only pre-existing variable influencing the IMM-AGE score was South Asian ethnicity ($\beta$ = 0.174, *p* = 0.043), with a major influence being disease severity ($\beta$ = 0.188, *p* = 0.01).

**Conclusions:**

Our analyses reveal a state of enhanced immune ageing in survivors of severe COVID-19 and suggest this could be related to SARS-Cov-2 infection. Our data support the rationale for trials of anti-immune ageing interventions for improving clinical outcomes in these patients with severe disease.

**Supplementary Information:**

The online version contains supplementary material available at 10.1186/s12979-023-00406-z.

## Background

The pandemic of coronavirus disease 2019 (COVID-19), arising from infection with the severe acute respiratory syndrome coronavirus 2 (SARS-CoV-2), has resulted in over 6 million deaths worldwide. SARS-CoV-2-infection exhibits a broad spectrum of disease manifestations ranging from mild symptoms such as fever, cough and fatigue to moderate and severe illness with radiological abnormalities detected on chest imaging [1]. Severe, life-threatening COVID-19 is further characterised by severe pneumonia requiring invasive and non-invasive respiratory support in intensive care units. Patients over 65 had the highest risk of severe disease and death with one meta-analysis of 70 studies suggesting the risk of in-hospital death increased by 5.7% per age year [2]. These data are similar to other viral illnesses, such as influenza, where older adults are more susceptible and have higher mortality [3]. An improved understanding of the impact of immune remodelling with age upon the severity of infectious disease in older adults may help us to identify therapeutic targets and better plan for future pandemics and seasonal viral infections. Further, if such remodelling persists beyond the acute infection period this could contribute to immune features seen in Long COVID such as persistent inflammation [4] and raised serum autoantibody levels [5].

The immune system is substantially remodelled with advancing age, termed immunesenescence [6], increasing susceptibility to infections, reducing vaccination responses and increasing the risk of autoimmunity [7]. The hallmarks of immunesenescence include an accumulation of CD56^dim^ cytotoxic NK cells with reduced cytotoxicity [8], thymic atrophy resulting in reduced naïve T cell output [9], accumulation of memory, exhausted and senescent T cells [10], skewing towards Th17 polarisation [11] and an expansion of regulatory T cells ( T_regs_) with an impaired suppressive capacity [12]. Like their non-immune counterparts, senescent T cells are pro-inflammatory with the characteristic senescence-associated secretory phenotype (SASP) of cytokines and chemokines [13]. Macrophages in older adults also have an inflammatory phenotype, producing a range of pro-inflammatory cytokines in the absence of infection [14]. These changes contribute to the pro-inflammatory status of older adults, so-called inflammageing. Advancing age is also accompanied by reduced B cell lymphopoiesis, resulting in a reduction of naïve and regulatory B cells and an accumulation of memory B cells [15, 16]. Marked elevation of pro-inflammatory cytokines such as interleukin (IL)-6, monocyte chemoattractant protein-1 (MCP-1), macrophage inflammatory protein-1 alpha (MIP-α), and tumour necrosis factor (TNF-α) is a key feature of severe COVID-19 disease [17], suggestive of a dysregulated immune response to infection which could include exaggerated immunesenescence.

Adaptive antiviral immunity includes the generation of antigen-specific cytotoxic CD8 T cells that effect the killing of virally infected cells, CD4 helper T cells that support antibody production by B cells and the generation of regulatory cells to ensure resolution of the response. In the acute phase of COVID-19, there is T cell lymphopenia with CD8 T cells displaying a hyperactivated phenotype, followed by the appearance of T cells with features of senescence and exhaustion [18]. Furthermore, a shift in T cell responses towards a pro-inflammatory Th17 phenotype [19] and altered composition of regulatory T cells [20] results in severe inflammation and respiratory system injury in COVID-19, with this Th17/T_reg_ imbalance associated with poor prognosis [21]. The number of Natural Killer (NK) cells, which also play a vital role in the clearance of viral infections, are reduced in the acute phase of COVID-19, and these cells also show features of senescence and functional impairment in severe disease [22]. Alterations have also been observed in B cells, including a reduction in naïve B cells and an elevation of plasmablasts in SARS-CoV2 infected patients compared to healthy controls [23]. Whether these features represent a state of heightened immunesenescence and may persist and underlie the severity of COVID-19 has not been established.

The current study aimed at using deep immunophenotyping to determine the degree of immunesenescence in convalescent SARS-CoV-2-infected individuals, 3–5 months after their recovery, comparing them to age and sex-matched SARS-CoV-2-unexposed participants (healthy controls). We measured a broad range of immune features in order to assess the impact on individual cell types but also to enable the generation of a composite score for immunesenescence, IMM-AGE. This score has been shown to relate to mortality in a longitudinal study of immune phenotype [24].

## Results

### Participant demographics and clinical characteristics

One hundred three adults with PCR-confirmed SARS-CoV-2 infection were recruited 3–5 months post-initial diagnosis. Fifty-six adults had COVID-19 classified as severe (age 53.12 ± 11.30 years; 31 males, 55%), thirty-two as moderate (age 52.28 ± 11.43 years; 15 males, 46%) and fifteen as mild (age 49.67 ± 7.30 years; 4 males, 26%) disease. There was no difference in the average age of the different disease severity groups, but males were a higher component in the moderate and severe groups compared to the mild disease group. There was also the highest frequency of patients from ethnic minority groups (34, 60%) in the severe disease group and the prevalence of patients with pre-existing multimorbidity was highest in the moderate (46%) and severe (58%) groups, with only one patient with multimorbidity in the mild group. Additionally, fifty-nine healthy age and sex-matched uninfected healthy controls (age 50.49 ± 10.68 years; 29 males, 47%) were recruited into the study (Table 1).

**Table 1:** Participant demographics and clinical parameters

	**Healthy controls (*n* = 59)**	**Mild COVID-19 (*n* = 15)**	**Moderate COVID-19 (*n* = 32)**	**Severe COVID-19 (*n* = 56)**	***p* value**
**Age ( mean ± SD)**	50.49 ± 10.68	49.67 ± 7.30	52.28 ± 11.43	53.12 ± 11.30	*p* = 0.50
**Males (%)**	28 (47%)	4 (26%)	15 (46%)	31 (55%)	*p* = 0.36
**ICU length of stay (days)**	0	0	0	19.18 ± 9.35	
**Ventilator days**	0	0	0	13.26 ± 8.66	
**Hospital length of stay**	0	0	10.25 ± 13.46	33.11 ± 13.86	*p* < .001
**Ethnicity (% Caucasian)**	18 (30%)	2 (13%)	6 (18%)	34 ( 60%)	*p* = 0.02
**Body Mass Index (kg/m**^2^**)**	31.47 ± 6.7	29.32 ± 5.4	31.83 ± 4.2	27.20 ± 3.2	*p* = 0.09
**Number of co-morbidities**					
***n*** **= 0**	59 ( 100%)	5 (33%)	10 (31%)	11 (19%)	
***n*** **= 1**	0	9 (60%)	7 (21%)	12 (21%)	
***n*** **= 2**	0	1 (7%)	6 (18%)	11 (19%)	
***n*** **= 2 + **	0	0	9 (28%)	22 (39%)	
**Smoking status**					
** Smoker**	0	0	2 (6%)	0	
** Non-smoker**	58 (98%)	15 ( 100%)	24 (75%)	54 (96%)	
** Ex-smoker**	1 (1.6%)	0	6 (18%)	2 (4%)	

### T cell phenotype in convalescent COVID-19 patients

**Fig. 1:**
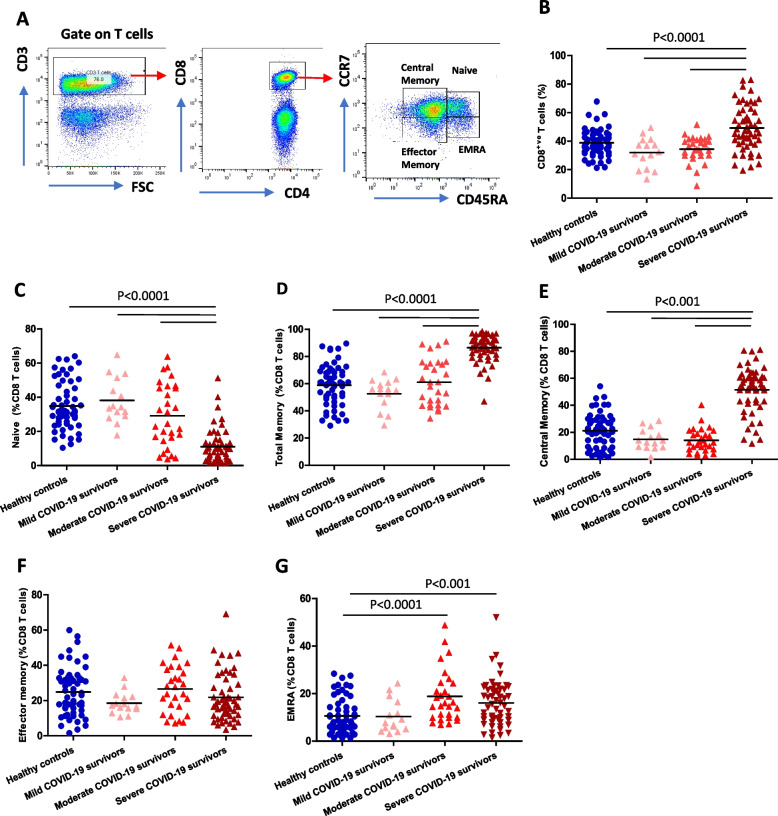
CD8 T cell subset distribution post-COVID-19 infection. **A** Gating strategy used to analyse markers subsets within CD4^+ve^ and CD8^+ve^ T cells; naïve (CCR7^+ve^CD45RA^+ve^); central memory (CCR7^+ve^CD45RA^−ve^), effector memory (CCR7^−ve^CD45RA^−ve^) and terminal differentiated effector memory re-expressing RA, EMRA (CCR7^−ve^CD45RA^+ve^) T cells. Comparison of the systemic percentage of: **B** CD8 T cells; **C** Naïve CD8 T cells; **D** Total memory CD8 T cells; **E** Central memory CD8 T cells; **F** Effector memory CD8 T cells **G** EMRA CD8 T cells. PBMCs were isolated from convalescent COVID-19 patients who had mild (*n* = 15), moderate (*n* = 29) and severe (*n* = 55) disease 3–5 months post-infection, and healthy age and sex-matched controls (*n* = 59). Data represent individual values, mean (centre bar). Statistical analysis by two-sided Mann–Whitney nonparametric test

For the immune phenotyping, we did not have cell count data for the mild disease group and so for this group only the cell frequencies are available. Firstly, we assessed CD4 and CD8 T cell subset distributions (Fig. 1A). Total T cell frequency in the PBMC fraction showed no significant differences between healthy controls and COVID-19 survivors of different disease severity, *F* (3,154) = 1.101, *p* = 0.35. Cytotoxic CD8 T cells play a vital role in immune defence against several viral infections, including coronavirus [25]. We observed an elevated frequency (Fig. 1B) and absolute numbers (Supplementary Fig. 1A) of CD8 T cells in severe COVID-19 convalescent patients in comparison with healthy controls, *p* < 0.0001 and *p* < 0.01, respectively. Within the CD8 T cell pool, the frequency (Fig. 1C) and number (Supplementary Fig. 1B) of naïve T cells were lower in severe COVID-19 survivors, both *p* < 0.0001. This was accompanied by an increased frequency (*p* < 0.0001, Fig. 1D) and absolute number, *p* < 0.001 (Supplementary Fig. 1C) of memory CD8 T cells in severe COVID-19 infection survivors. Amongst the CD8 T cell memory pool, there was an increase in frequency (*p* < 0.001, Fig. 1E) and absolute numbers (*p* < 0.001, Supplementary Fig. 1D) of central memory CD8 T cells in severe COVID-19 patients. We did not observe an increase in the frequency (*p* = 0.29, Fig. 1F) or absolute numbers (*p* = 0.65, Supplementary Fig. 1E) of effector memory CD8 T cells in severe COVID-19 patients, but we did see increases in terminally differentiated EMRA CD8 T cells in both moderate (*p* < 0.0001) and severe (*p* = 0.001) COVID-19 patients (Fig. 1G). An expansion of absolute EMRA numbers was only observed in the severe disease cohort (*p* < 0.001, Supplementary Fig. 1F).

**Fig. 2:**
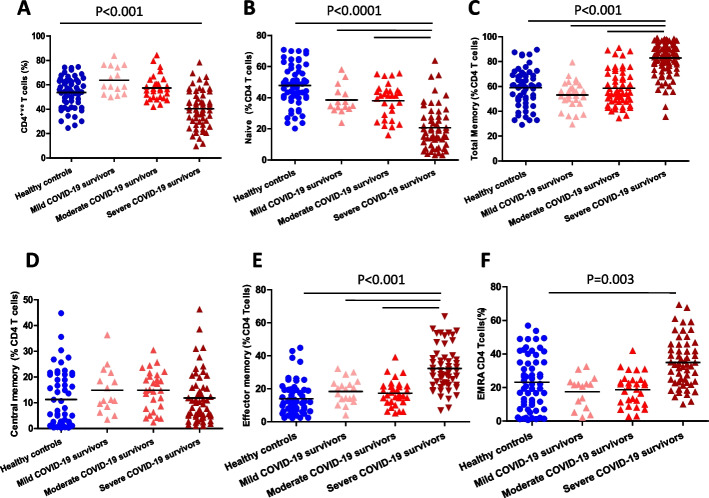
CD4 T cell distribution post-COVID-19 infection. Comparison of the systemic percentage of (**A**) CD4 T cells; **B** Naïve CD4 T cells; **C** Total memory CD4 T cells; **D** Central memory CD4 T cells; **E** Effector memory CD4 T cells (F) EMRA CD4 T cells. PBMCs were isolated from convalescent COVID-19 patients who had mild (*n* = 15), moderate (*n* = 29) and severe (*n* = 55) disease 3–5 months post-infection, and healthy age and sex-matched controls (*n* = 59). Data represent individual values, mean (centre bar). Statistical analysis by two-sided Mann–Whitney nonparametric test

In contrast to the CD8 T cell population, we observed a lower frequency (*p* < 0.001, Fig. 2A) and absolute cell number, (*p* < 0.001, Supplementary Fig. 2A) for CD 4 T cells in severe COVID-19 patients. Within the CD4 T cell pool there was a significantly lower frequency (*p* < 0.0001, Fig. 2B) and absolute number (*p* < 0.0001, Supplementary Fig. 2B) of naïve CD4 T cells in the severe COVID-19 cohort. This was accompanied by an increase in the frequency (*p* < 0.001, Fig. 2C) but not absolute number (*p* = 0.19, Supplementary Fig. 2C) of memory CD4 T cells. Amongst memory CD4 T cells, no differences were observed in frequency [*p* = 0.31, Fig. 2D] and absolute numbers (*p* = 0.42, Supplementary Fig. 2D) of central memory CD4 T cells. However, there was an increase in the frequency (*p* < 0.001, Fig. 2E) and absolute numbers (*p* = 0.001, Supplementary Fig. 2E) of effector memory and the frequency of the EMRA population (*p* = 0.003, Fig. 2F), but not absolute numbers ( Supplementary Fig. 2F).

### COVID-19 and T cell senescence and exhaustion

**Fig. 3:**
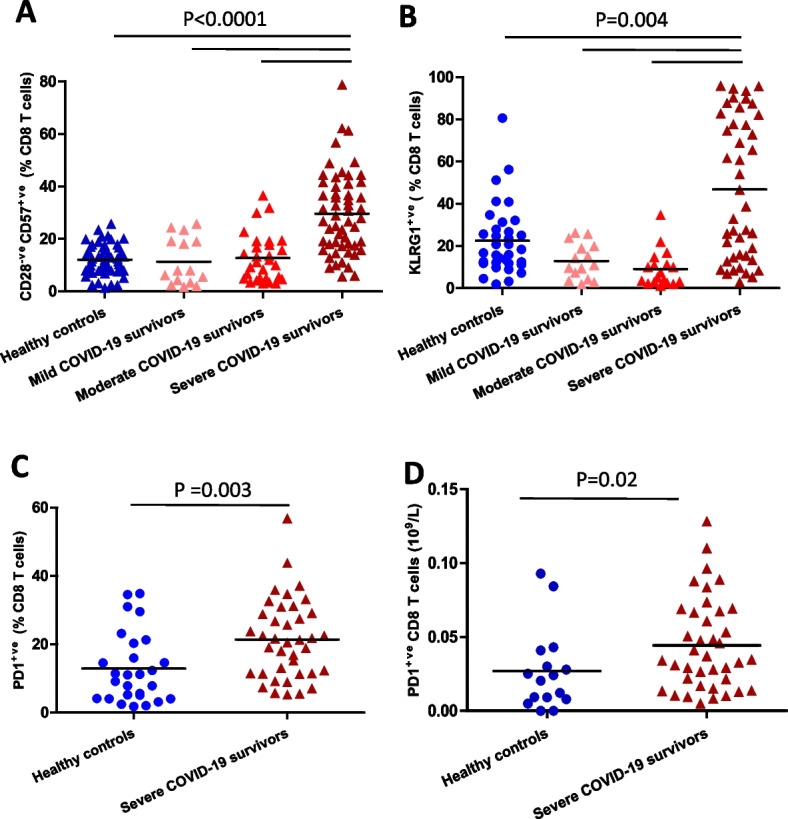
CD8 T cell senescence and exhaustion post-COVID-19 Comparison of systemic percentage of (**A**) CD28^−ve^ CD57^+ve^ senescent CD8 T cells in healthy age and sex-matched controls (*n* = 59) and mild (*n* = 15), moderate (*n* = 29) and severe (*n* = 55) COVID-19 survivors 3–5 months post-infection. **B** Frequency of KLRG1^+ve^ senescent CD8 T cells in healthy age and sex-matched controls (*n* = 51) and mild (*n* = 15), moderate (*n* = 24) and severe (*n* = 46) COVID-19 survivors 3–5 months post-infection. **C** percentage and (**D**) absolute numbers of PD1^+ve^ exhausted CD8 T cells in healthy age and sex-matched controls (*n* = 33) and severe (*n* = 38) COVID-19 survivors 3–5 months post-infection. Statistical analysis by two-sided Mann–Whitney non-parametric test. If not indicated *p*-valueue is not significant

T cells can be further subdivided based on the expression of the co‐stimulatory molecule CD28, which is lost as they differentiate to an effector phenotype and subsequently gain expression of markers, such as CD57 [26] and Killer cell lectin-like receptor subfamily G member 1 (KLRG1) [27] as they become senescent. Severe COVID-19 convalescent patients had a higher frequency (*p* < 0.0001, Fig. 3A) and absolute numbers (*p* < 0.001, Supplementary Fig. 3A) of CD28^−ve^ CD57^+ve^ CD8 T cells, in comparison with healthy controls and mild and moderate convalescent patients. A similar accumulation of CD28^−ve^ CD57^+ve^ CD4 T cells was seen in severe COVID-19 convalescent patients (Supplementary Fig. 3B, C). We saw an expansion in the frequency of CD8 T cells also expressing KLRG1 (*p* = 0.004, Fig. 3B), but this did not equate to an increase in absolute numbers (Supplementary Fig. 3D). No increase was observed in the KLRG1 expressing CD4 T cell pool (Supplementary Fig. 3E, F). Next, we investigated markers of T cell exhaustion, specifically PD-1 expression [28]. We found an increase in frequency (*p* = 0.003, Fig. 3C) and numbers [*p* = 0.02, Fig. 3D) of PD1 expressing CD8 T cells in severe COVID-19 convalescent patients. A similar state of expansion of exhausted cells was not observed in the CD4 T cell pool of severe COVID-19 patients (Supplementary Fig. 3G, H).

### COVID-19 and CD4 helper T cell subset distribution: T_reg_ and Th17 cells

**Fig. 4:**
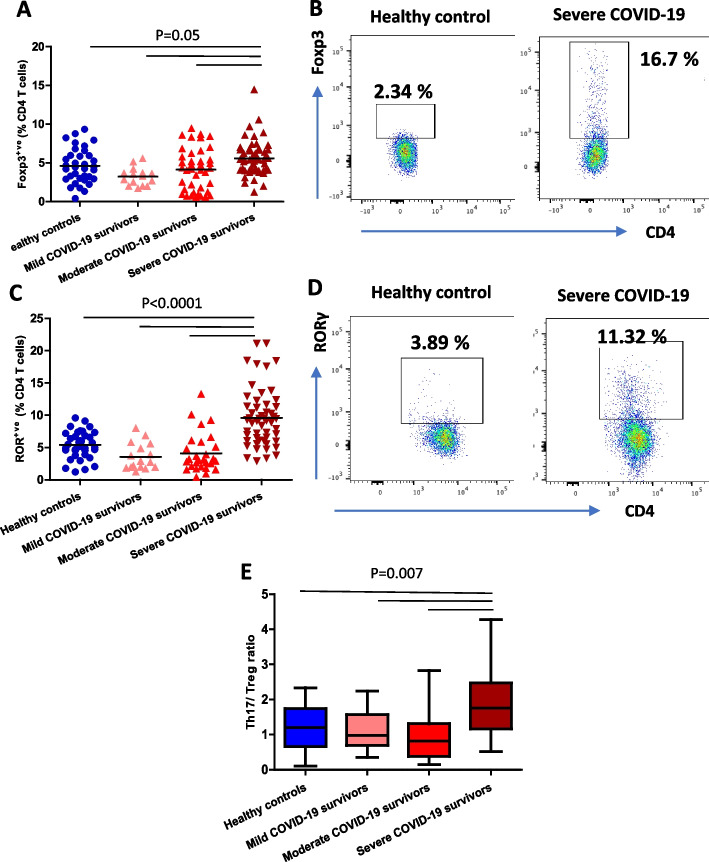
The impact of COVID-19 on Regulatory T cells and Th17 cells. **A** Comparison of systemic percentage of Foxp3^+ve^ CD4 T cells in healthy age and sex-matched controls (*n* = 59) and mild (*n* = 15), moderate (*n* = 29) and severe (*n* = 55) COVID-19 survivors 3 months post-infection. **B** Representative flow cytometry plot showing Foxp3^+ve^ regulatory T cells in a healthy control and severe convalescent COVID-19 patients. **C** Comparison of systemic percentage of RORγt^+ve^ CD4 T cells in healthy age and sex-matched controls (*n* = 59) and mild (*n* = 15), moderate (*n* = 29) and severe (*n* = 55) COVID-19 survivors 3–5 months post-infection. **D** Representative flow cytometry plot showing RORγt^+ve^ Th17 cells in a healthy control and severe convalescent COVID-19 patient. **E** Th17/T_reg_ ratio in healthy age and sex-matched controls (*n* = 59) and mild (*n* = 15), moderate (*n* = 29) and severe (*n* = 55) COVID-19 survivors 3–5 months post-infection. Statistical analysis by two-sided Mann–Whitney non-parametric test. If not indicated, *p* value is not significant

Naïve CD4^+^ T cells differentiate into several functional types of effector cells with distinct cytokine secretory profiles. Foxp3^+ve^ CD4 T cells have been classified as regulatory T cells, T_reg_, that control the magnitude of immune responses and suppress excessive inflammation [29] and their numbers increase with age. Here we observed a modest expansion in the frequency of T_reg_ cells in the severe COVID-19 convalescent cohort [*p* = 0.05 (Fig. 4A, B). RAR-related orphan receptor (ROR)γt expressing Th17 cells, produce IL-17A, IL-17F, IL-21 and IL-22, which play a crucial role in driving inflammation during the pathogenesis of inflammatory disorders [30]. In this study we detected a two-fold expansion in the Th17 population in severe COVID-19 convalescent patients (*p* < 0.0001, Fig. 4C, D). These changes resulted in an increased Th17/T_reg_ ratio in severe COVID-19 patients (*p* = 0.007, Fig. 4E), indicating a CD4 compartment that is skewed towards a more pro-inflammatory phenotype.

### COVID-19 and B cell subset distribution

**Fig. 5:**
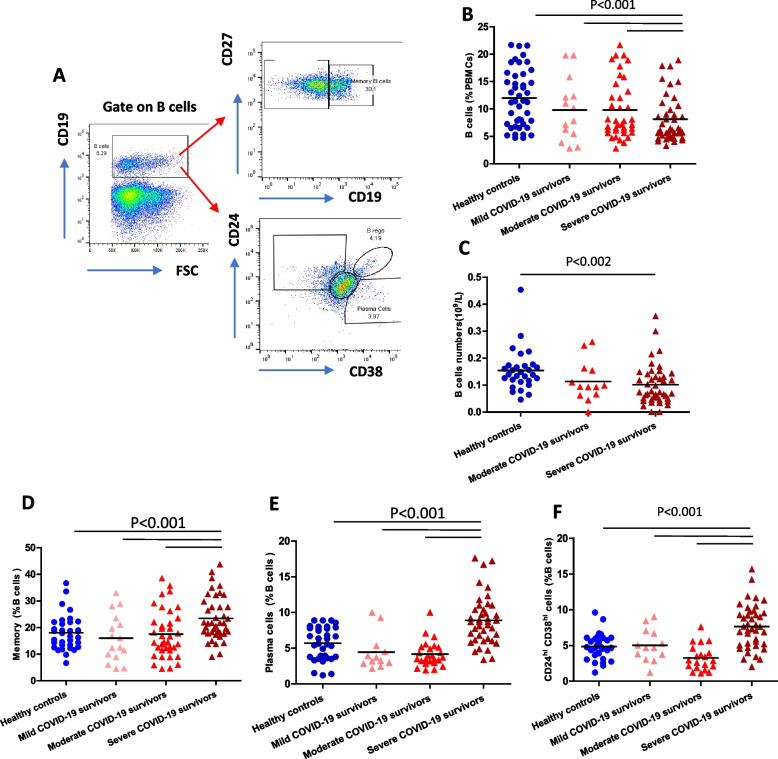
B cell subset distribution post-COVID-19. **A** Gating strategy used to analyse subsets within CD19^+ve^ B cells; naïve (CD27^−ve^); memory (CD27^+ve^), regulatory B cells (CD38^hi^CD24^hi^) and plasma cells (CD24^−ve^CD38^+ve^) B cells. **B** Comparison of systemic percentage of total B cells in healthy age and sex-matched controls (*n* = 59) and mild (*n* = 15), moderate (*n* = 29) and severe (*n* = 55) COVID-19 survivors 3–5 months post-infection. **C** Absolute numbers of B cells in healthy age and sex matched controls (*n* = 39), moderate (*n =* 14) and severe (*n* = 46) COVID-19 convalescent patients. Comparison of systemic percentage of (**D**) memory B cells, (**E**) Plasma cells, (**F**) regulatory B cells in healthy age and sex-matched controls (*n* = 59) and mild (*n* = 15), moderate (*n* = 29) and severe (*n* = 55) COVID-19 survivors 3–5 months post-infection. Statistical analysis by two-sided Mann–Whitney non-parametric test. If not indicated, p-value is not significant

We also investigated if convalescent individuals who have experienced mild, moderate and severe COVID-19 had perturbed B cell populations (Fig. 5A). Firstly, we observed a significantly lower frequency (*p* < 0.001, Fig. 5B) and number (*p* = 0.002, Fig. 5C) of B cells only in severe COVID-19 patients in comparison with healthy controls. Within the B cell pool, there was a significant expansion in the frequency (*p* < 0.001, Fig. 5D) of memory B cells. There was also an expansion in the frequency of CD38^hi^ terminally differentiated plasma B cells (*p* < 0.001, Fig. 5E), an essential source of protective antibodies. In addition to antibody production, a subset of B cells, known as regulatory B cells (Breg), exhibit immunosuppressive functions via the secretion of IL‐10 and tumour growth factor‐β (TGF‐β). This population has recently gained attention for their critical role in the maintenance of immune homeostasis and ability to suppress Th17 responses [31]. Similar to regulatory T cells, we observed a higher frequency of CD24^hi^ CD38^hi^ regulatory B cells within the B cell pool (*p* < 0.001, Fig. 5E). However, the absolute numbers of these B cell subsets did not differ significantly in the COVID-19 patients compared to the controls (Supplementary Fig. 4A-C).

### COVID-19 and NK cell phenotype

**Fig. 6:**
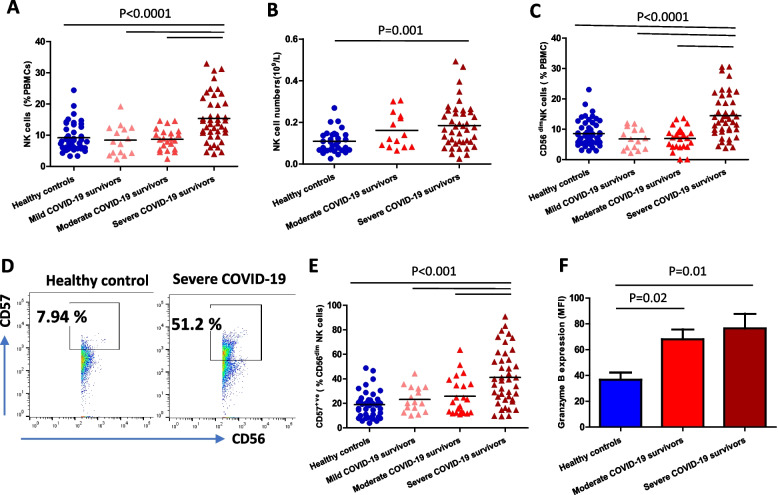
NK cells in severe COVID-19 convalescent patients. **A** Comparison of the systemic percentage of total NK cells in healthy age and sex-matched controls (*n* = 59) and mild (*n* = 15), moderate (*n* = 29) and severe (*n* = 55) COVID-19 survivors 3–5 months post-infection. **B** Absolute numbers of NK cells in healthy age and sex matched controls (*n* = 39), moderate (*n* = 14) and severe (*n* = 46) COVID-19 convalescent patients. **C** Comparison of the systemic percentage of CD56^dim^ cytotoxic NK cells in healthy age and sex-matched controls (*n* = 59) and mild (*n* = 15), moderate (*n* = 29) and severe (*n* = 55) COVID-19 survivors 3–5 months post-infection. **D**, **E** Comparison of the systemic percentage of senescent NK cells in CD56 dim NK cell pool healthy age and sex-matched controls (*n* = 59) and mild (*n* = 15), moderate (*n* = 29) and severe (*n* = 55) COVID-19 survivors 3–5 months post-infection (F) Granzyme B expression levels in NK cells in healthy age and sex-matched controls (*n* = 29) and severe (*n* = 31) COVID-19 convalescent patients. Statistical analysis by two-sided Mann–Whitney non-parametric test. If not indicated, *p* value is not significant

Natural killer (NK) cells are innate lymphoid cells that play a key role in providing protection from viral infections. Their numbers increase with age but their cytotoxicity declines. Here we observed a higher frequency (*p* < 0.0001, Fig. 6A) and absolute number (*p* = 0.001, Fig. 6B) of NK cells in severe COVID-19 convalescent patients. NK cells can be divided into two subsets based of the expression of CD56: cytokine-secreting CD56^bright^ and cytotoxic CD56^dim^ cells [32]. We found that the increase in NK cells with severe disease was driven by an accumulation of cytotoxic CD56^dim^ cells (*p* < 0.0001, Fig. 6C). CD57 expression defines a functionally discrete sub-population of terminally differentiated and functionally senescent NK cells [33]. We detected a higher frequency (*p* < 0.001, Fig. 6D, E) and absolute number (*p* = 0.004, data not shown) of CD57^+ve^ CD56 ^dim^ NK cells in severe COVID‐19 convalescent patients compared to healthy controls. To characterise NK cell cytotoxic potential further we performed intracellular staining for the expression of the cytotoxic enzyme granzyme B (GzmB) [34]. Surprisingly Granzyme B expression in NK cells of COVID-19 patients was significantly elevated in moderate (*p* = 0.02) and severe disease cohorts (*p* = 0.01) in comparison with healthy controls (Fig. 6F).

### COVID-19 and IMM-AGE scores

**Fig. 7:**
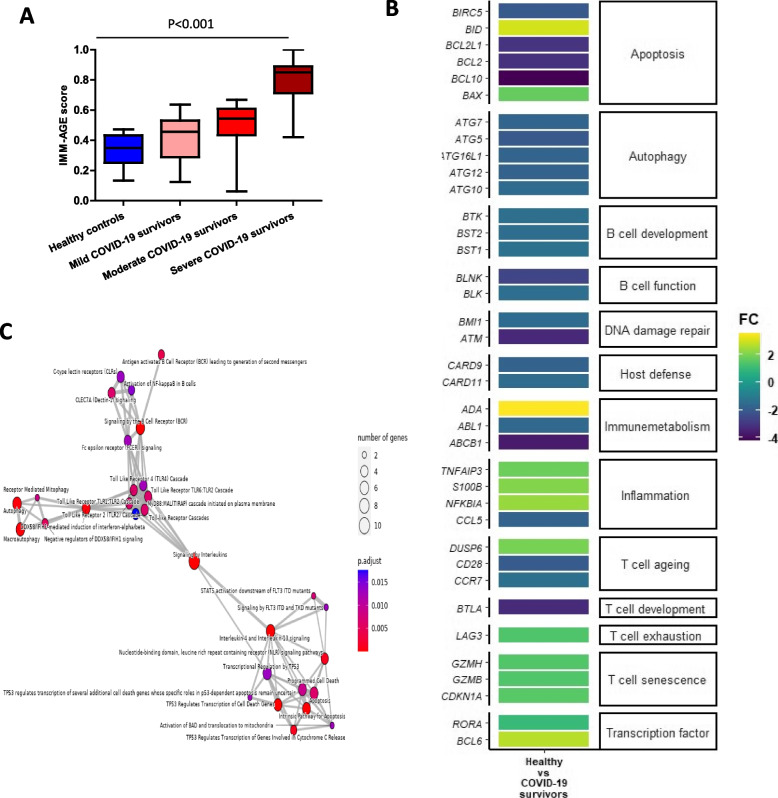
Immunological ageing score (IMM-AGE) and transcriptome signatures in severe COVID-19 convalescent patients. **A** IMM-AGE scores calculated by the pseudotime algorithm^23^ in healthy age and sex-matched controls (*n* = 39) and mild (*n* = 15), moderate (*n* = 33) and severe (*n* = 42) COVID-19 survivors 3–5 months post-infection. Statistical analysis by two-sided Mann–Whitney non-parametric test. If not indicated, *p* value is not significant. **B** (**B**) A heatmap showing the relative expression levels of a selection of significantly differentially expressed genes between the healthy control and severe COVID-19 groups. The gene IDs can be seen on the X axis. The figure legend colour corresponds to the relative expression levels of a given gene within a group. **C** An map plot showing the relationships between the pathways associated with the set of significantly differentially expressed genes between healthy control participants and survivors of severe covid-19 infection. Node size denotes the number of genes associated with a specific pathway, with increasing size reflecting a greater number, and colour reflects the adjust p-valuealue

IMM-AGE is a recently developed metric, consisting of 20 T cell subset frequecnies. IMM-AGE describes an individual’s cellular immune profile in relation to their chronological age and has been recognised as a reliable predictor of all-cause mortality in older adults [24]. Here we used a modified version that requires only 8 T cell subsets (total T cells, naive CD4 T cells, effector memory CD4 and CD8 T cells, EMRA CD8 T cells, CD28^−ve^ CD8 T cells, CD57^+ve^ CD8 T cells and regulatory T cells) [35]. Compared to healthy controls, we observed a significantly higher IMM-AGE score in patients who had had severe COVID-19 (*p* < 0.001, Fig. 7A), the higher scores seen in mild and moderate disease did not reach significance. To try and understand to what extent the higher IMM-AGE scores reflected pre-existing immunesenescence, or were the result of COVID-19, we carried out multiple linear regressions considering variables that could affect the score namely BMI, multimorbidity, ethnicity, smoking status and sex (Table 2). The analysis revealed the only pre-existing variable influencing the IMM-AGE score was South Asian ethnicity ($\beta$ = 0.173, *p* = 0.041), with the major influence being disease severity ($\beta =$ 0.187, *p* = 0.01).

**Table 2:** Multiple linear regressions of IMM-AGE score with participant demographics and clinical parameters

Coefficients	Std Error	T value	Pr ( >|t|)
Ethnicity_Black	0.0829047	0.191	0.8499
Ethnicity_Asian	0.0945755	-2.452	0.0222
Ethnicity_Caucasian	0.0482719	-2.114	0.0456
Age	0.0042638	0.16	0.8745
Gender_male	0.0526779	0.653	0.5205
BMI	0.0036713	-1.601	0.123
Number of co_morbidities	0.0448307	-1.489	0.15
Smoking status_yes	0.1205799	-0.231	0.8194
Smoking status_no	0.0736119	-0.488	0.6301
ICU_Length of stay	0.0046717	-0.542	0.5931
Length of stay	0.0027478	0.837	0.4115
Ventilatory days	0.0025575	1.646	0.1133

### Transcriptome signature of severe COVID-19 convalescent patients

To elucidate molecular signalling pathways in peripheral immune cells that might contribute toward this state of enhanced immune ageing in severe COVID-19 patients we used the Nanostring nCounter gene expression assay. To obtain a homogenous cohort for the gene expression analysis all ten participants [5 severe COVID-19 survivors and 5 healthy controls] are Caucasian non-smokers with a healthy BMI and no underlying co-morbidities. The Healthy control participants have been closely age and gender-matched with the COVID-19 survivors cohort. This allowed for the detection of 770 genes in PBMCs from five Caucasian convalescent severe COVID-19 patients and five healthy controls. Atable showing the mean gene expression data (Table 3) and a heatmap showing fold change of the 38 differentially expressed genes (Fig. 7B). The analysis confirmed the flow cytometry data suggesting a more senescent or exhausted phenotype, with a reduction in expression of CD28 and CCR7 and an upregulation in the exhaustion marker (LAG3), the transcription factor involved in Th17 polarisation (RORA) and cytotoxic Granzymes B and H which are upregulated in senescent cells]. On conducting an enrichment analysis of these genes, the most enriched pathways included ageing-related pathways such as inflammation, cellular senescence, apoptosis and autophagy (Fig. 7C). A downregulation of genesinvolved in DNA damage repair signalling (eg Ataxia-telangiectasia mutated ATM) [36] also suggests a more aged phenotype, though reduced cyclin-dependent kinase inhibitor p21 (CDKN1A) would not suggest a fully proliferatively senescent phenotype [37, 38]. Autophagy a key cellular process of clearance of damaged organelles and macromolecules has been shown to be reduced in T cells from aged donors, contributing to immunesenescence [39]. Here we found downregulation of five autophagy-related genes (including Atg7, Atg5) in PBMCs of severe COVID-19 convalescent patients. Furthermore, we found that significantly expressed genes were involved in inflammation (e.g. NF-kB signalling, *TNFAIP3 and pro-inflammatory chemokine CCL5,* S100 calcium binding protein B (S100B)), anti-fungal immunity (eg CARD-9) and B cell development/function (e.g. B-cell lymphocyte kinase (Blk) pathways and intrinsic pathways of apoptosis (e.g. downregulation of ati-apoptotic bcl2, upregulation of pro-apoptotic BID) (Fig. 7B).

**Table 3:** Mean gene expression levels of differentially expressed genes between healthy controls and severe COVID-19 survivors

Gene	Healthy Controls	COVID-19 survivors	*p* value
*BCL2*	278.99	67.28	0.03
*BCL2L1*	276.93	271.94	0.03
*BID*	29.28	54.7	0.03
*BIRC5*	21.03	24.69	0.03
*BAX*	361.4	428.67	0.02
*BCL10*	637.5	642.26	0.03
*ATG10*	39.56	25.06	0.02
*ATG12*	25.81	21.35	0.02
*ATG16L1*	194.77	178.78	0.02
*ATG5*	206.18	219.66	0.02
*ATG7*	211.81	75.37	0.02
*BTK*	289.14	169.69	0.04
*BST1*	578.34	395.11	0.03
*BST2*	208.86	144.52	0.03
*BLK*	26.86	20	0.03
*BLNK*	46.15	22.66	0.03
*ATM*	29.97	20	0.02
*BMI1*	123.69	134.08	0.03
*CARD11*	398.91	269.75	0.05
*CARD9*	107.22	39.17	0.05
*ABCB1*	158.99	133.05	0.001
*ABL1*	108.46	108.43	0.001
*ADA*	251.94	289.47	0.001
*NFKBIA*	3981.45	8074.45	0.05
*TNFAIP3*	1438.51	3295.49	0.05
*CCL5*	776.86	1562.88	0.05
*S100B*	20	71.49	0.04
*DUSP6*	1108.61	1723.26	0.04
*CCR7*	199.46	28.56	0.05
*CD28*	109.88	58.2	0.05
*BTLA*	189.81	47.55	0.04
*LAG3*	54.16	92.48	0.05
*CDKN1A*	305.75	514.31	0.04
*GZMB*	1595.35	2154.99	0.03
*GZMH*	393.31	701.41	0.03
*RORA*	497.25	645.93	0.05
*BCL6*	188.63	275.4	0.03

## Discussion

COVID-19, in common with other severe respiratory conditions [2], is associated with greater morbidity and mortality in older adults [3]. One of the potential explanations is that the ageing of the immune system makes older adults more susceptible to these infections, less well able to control them and more prone to harmful responses such as hyperinflammation and autoimmunity. Equally likely is the possibility that the infection itself would increase features of immunesenescence which may be acute or persistent. In the acute phase of infection, studies have shown evidence of an exhausted as well as an aged immune phenotype in COVID-19 patients, such as CD8 T cells and NK cells with reduced IL-2 and IFN-γ expression, reduced granzyme expression and degranulation (CD107a) and an increased expression of the inhibitory receptor NKG2A [40, 41]. Moreover, this phenotype was more prominent with increasing disease severity suggesting that it may have influenced the compromised response to infection. Here we have taken these observations of individual cell phenotype changes forward and used a composite score of immune ageing, IMM-AGE [24, 35], to determine any association of immunesenescence with COVID-19 disease severity. We also recruited patients 3–5 months post-diagnosis to try and eliminate the influence of changes to immune cell profile in the acute phase. Our data reveal a greater degree of immune ageing, demonstrated by a higher IMM-AGE score, which was only seen in those with severe disease, though a trend to a higher score was also seen with moderate disease.

A key question addressed in our study was whether this higher degree of immunesenescence was present prior to infection, or was driven by the infection. Supporting the argument in favour of patients with severe disease potentially having a more aged immune system prior to infection is a study of participants in UK Biobank. For 347,571 individuals it was possible to calculate how biologically old they were when they enrolled in the study between 2006 and 2010, as opposed to their chronological age, using blood biochemistry data to derive the PhenoAge score. The analysis revealed that those participants who went on to develop severe COVID-19 were 10–14 years older biologically [42]. Crucially, in our study the prevalence of patients with pre-existing multimorbidity was highest in the moderate (46%) and severe (58%) groups, with only one patient with multimorbidity in the mild group. As the IMM-AGE algorithm was developed from longitudinal data and mortality [24] we would predict a higher score in a group at higher risk of death, i.e. those with multimorbidity or a high BMI [43]. However our multiple linear regression model revealed that neither multimorbidity nor BMI contributed significantly to the IMM-AGE score, instead South Asian ethnicity contributed to 17% of the increase in the IMM-AGE score. Interestingly we have shown recently that South Asian adults develop a broad range of immune-mediated diseases much earlier than white adults, possibly suggesting that their immune systems age more rapidly [44]. Our data thus suggest that the SARS-CoV2 infection itself increases immune ageing. The antigenic stimulation occurring during viral infections will certainly lead to telomere shortening, the appearance of more highly differentiated EMRA T cells, as well as exhausted and senescent T cells [45]. A similar state of acceleration of immune ageing has been observed in our studies in a younger cohort of traumatic injury patients [35], suggesting a negative influence of an acute challenge to immunesenescence. Our regression analysis revealed that the severity of disease made a significant contribution to the IMM-AGE score, supporting a major association of the SARS-CoV2 infection with immunesenescence.

Whether or not this enhanced immunesenescence is a result or consequence of COVID-19, it does suggest that these patients will be more vulnerable to future infections, show compromised vaccine responses and be at a higher risk of autoimmune disease. Moreover, as the induction of an aged immune system, specifically senescent CD4 T cells, has been shown in mice to be sufficient to drive an aged phenotype, including frailty and multimorbidity [46], our data may also suggest broader implications for the health of COVID-19 survivors. Evidence from recent studies has suggested the persistence of a spectrum of COVID-19 symptoms for up to 12 months after diagnosis, termed Long COVID, including persistent fatigue, myalgia and respiratory complications [47, 48]. Studies of COVID-19 convalescents 3–5 months post-infection have revealed maintained high levels of IL-6 associated with persistence of symptoms [4] and a study of autoantibody levels in serum found a high frequency of antibodies against the skin, skeletal muscle and cardiac tissue [5]. The aged immune system may thus contribute to both the acute and chronic sequelae of COVID-19, but we were unable to collect any information on post-acute sequellae of SARS-CoV-2 (PASC) in this cohort.

T cell lymphopenia has been widely reported during the acute phase of COVID-19 infection [49] and a small study investigating the T cell profile in a cohort of 13 convalescent patients, four weeks post-resolution of infection observed a loss of naïve CD4 T cells and accumulation of memory T cells [50]. Here we show that a numerical deficit of CD4 T cells persists in severe cases several months post-infection, particularly in the naïve T cells. Previous studies have reported that infections can result in thymic atrophy and changes in thymocyte development [51], a potential explanation for the reduction in naïve T cells. The potential consequence is a reduced ability to respond to new pathogens, including substantially different SARS-CoV-2 variants and reactivation of latent viruses (e.g. EBV and herpes). In contrast, we observed an expansion of CD8 T cells with a central memory phenotype, which could provide long-term effective memory responses. Whilst previous studies in SARS infection found that central memory T cell responses persist for up to 4 years post infection [52], a recent study of 188 patients has shown that memory CD4 and CD8 T cell numbers decline with a half-life of 3–5 months [53].

In patients who had severe COVID-19 the CD8 T cell profile features an increase in cells with a phenotype of senescence (defects in proliferation) and functional exhaustion, in agreement with previous reports from the acute phase [40, 41], suggesting that this is not a transient phenomenon. This observation not only raises concerns about the cytotoxic function of memory CD8 cells, but has broader consequences for health as senescent T cells are characterised by the secretion of a range of pro-inflammatory cytokines, chemokines, proteases and growth factors, termed the senescence-associated secretory phenotype (SASP) [54]. Thus, we hypothesise that the expansion of senescent T cells could be contributing towards the persistence of a pro-inflammatory environment in convalescent patients and symptoms such as fatigue and myalgia [48]. Another potential contributor to this inflammatory environment is the Treg/Th17 imbalance and increase in senescent CD57 expressing NK cells that we found also persisted in severe COVID-19 infection survivors several months post-acute infection. In support of this proposal, a study comparing the circulating immune profile of COVID-19 patients found an accumulation of senescent NK cells, Th17 cells and senescent T cells to be predictors for residual lung lesions [55]. The aged profile in the severe COVID-19 convalescent patients might therefore be contributing to impaired lung function and pulmonary fibrosis seen in some convalescent patients [56].

In addition to T cells, humoral immunity also plays a critical role in responding to viral infections and immunological B cell memory generated after infection is fundamentally important for protecting the host from severe disease upon re-exposure. In this study, we found reduced B cell numbers in convalescent patients irrespective of disease severity, but an expansion in the proportion of memory B cells and plasmablasts in severe COVID-19 patients. Multiple studies have detected virus-specific antibodies several months post-recovery from COVID-19, potentially a result of an elevated frequency of antibody-secreting plasmablasts [57, 58]. Furthermore, we observed an expansion of regulatory B cells, a subtype that produces IL10, in patients who had severe disease, which might be a compensatory mechanism for the expansion of pro-inflammatory immune cell subsets. These findings agree with another study reporting higher levels of IL10^+ve^ B cells in convalescent patients [59].

Although the detailed mechanisms driving a relationship between an aged immune phenotype and COVID-19 severity remain poorly understood, our RNA expression analysis has identified elevated inflammatory signalling, cellular senescence pathways and defects in DNA damage repair and autophagy, which are key processes underlying immunesenescence [60]. As the clinical consequences of immunesenescence include an increased risk of bacterial infections, reactivation of latent viruses, poor vaccine responses, increased risk of chronic inflammatory conditions [61, 62] and organ functional decline [63], severe COVID-19 survivors can thus be considered as a vulnerable population. Finding ways to alleviate immunesenescence should therefore be a priority to improve the health outcomes of these patients. Focusing on restoring thymic function could be considered a potential holistic treatment for rejuvenating the adaptive immune system and restoring immune homeostasis. The TRIIM (Thymus Regeneration Immunorestoration and Insulin Mitigation) trial has shown it is possible to boost thymic regeneration in older males using three agents: metformin, growth hormone and dehydroepiandrosterone given for 12 months [64]. An alternative method using an injection of Thymosin alpha 1(Tα1), known to support T cell generation and survival, reversed T cell exhaustion by boosting thymic output and reducing mortality in severe COVID-19 patients [65]. Another drug with anti-immunesenescence properties is metformin [66], which has been shown recently to reduce mortality in hospitalised COVID-19 patients [67]. Autophagy-boosting therapies, such as spermidine supplementation, have yielded promising results in rejuvenating an aged immune system in older adults [68]. Exercise has also been shown to induce its beneficial effects on body systems via the stimulation of autophagy [69]. Both interventions may therefore be useful in COVID-19 convalescent patients.

Our study has some limitations which should be considered when interpreting the findings. Firstly, we have only assessed immunological phenotype in convalescent patients and do not have longitudinal data from during the acute phase of infection, or prior to infection. We cannot therefore determine the degree to which the enhanced immune ageing was a cause or consequence of infection, though our regression analysis only found one pre-existing variable to influence the IMM-AGE score, namely South Asian ethnicity. Second, due to the collection of a limited volume of blood from the participants, it has not been possible to assess immune cell function in convalescent patients. However, our data does hint toward proliferative defects, skewing towards an inflammatory phenotype and TCR signalling defects due to overexpression of dual-specific phosphatase DUSP6, a feature of aged T cells that attenuates ERK signalling after TCR activation [70]. Third, by the very definition of healthy, our uninfected controls did not have any chronic disease and so were not well matched for the moderate and severe disease groups which had a substantial number of multimorbid patients. However, our regression analysis was able to show that the presence of multimorbidity was not a significant influence on immunesenescence. Additionally, there was variation in ethnicity amongst our four cohorts and this will have affected the data as the regression analysis revealed that South Asian ethnicity was an influence on the degree of immunesenescence.

## Conclusions

In summary, we have demonstrated a state of persistent enhanced immune ageing in adults during convalescence from severe COVID-19, potentially contributing to increased susceptibility to ongoing and future ill health in these patients. Our data support the rationale for trials of anti-immune ageing interventions for improving clinical outcomes in these patients.

## Methods

### Participants

This observational cohort study recruited adults with confirmed SARS-CoV-2 infection who were 3–5 months post-infection and age and sex-matched controls who had not been infected. Hospitalised patients were stratified into two groups based on their fraction of inspired oxygen (FiO2) levels and the need for respiratory support. Patients requiring between 28–60% FiO2 were classified as ‘moderate’ and those above 60% FiO2, or requiring admission to intensive care were classified as ‘severe’. The mild patients had polymerase chain reaction (PCR) confirmed SARS-CoV-2 infection but were not hospitalised. The screening, recruitment, and sampling took place at three sites in the UK: the Queen Elizabeth Hospital, Birmingham, University Hospitals of Leicester NHS Trust, and the University of Liverpool. Additional clinical measures, including ventilator days, length of ICU stay, and length of hospital stay were also recorded. The age and sex-matched healthy controls were students and staff at the University of Birmingham and older adults recruited from the community. Healthy or COVID-19 survivors were excluded if they had a self-reported infection at the time of sampling or a pre-existing immune-mediated disease. The severe COVID patients were recruited in Birmingham as part of the Coronavirus Immunological Analysis study approved by North West Preston Research Ethics Committee (20/NW/0240). The moderate disease patients were recruited as part of the PHOSP-COVID study approved by Leeds West Research Ethics Committee (20/YH/0225) and the Human Immune Responses to Acute Virus Infections study (16/NW/0170) approved by North West—Liverpool Central Research Ethics Committee. The mild disease cohort were recruited as part of the COVID in the Community study approved by the London Camden & Kings Cross Research Ethics Committee (20/HRA/1817).

### Blood cell isolation

Blood samples were collected by venepuncture into vacutainers containing heparin (Sastedt AG, Germany). Complete blood differential counts were performed in whole blood using a haematology analyser (Sysmex XN-1000, Sysmex, Germany). Whole blood count data for mild COVID-19 infection patients were unavailable as they were not hospitalised. Peripheral blood mononuclear cells (PBMCs) were isolated by density centrifugation using Ficoll-Paque™ PLUS (GE Healthcare, UK) of diluted blood (1:1) in RPMI 1640 medium (Sigma Aldrich, Poole, UK), and overlayered blood was centrifuged for 30 min at 400 × g at 20 °C without brake [71]. Isolated PBMCs were frozen by resuspending cells in a freezing medium consisting of 10% DMSO (Sigma Aldrich) in heat-inactivated fetal calf serum (FCS; Biosera, UK) and stored at -80°C until further analysis.

### T and B cell phenotyping

Frozen PBMCs were thawed at 37°C and washed in RPMI1640 containing 10% FCS prior to resuspension in phosphate-buffered saline (PBS) at 1 × 10^6^ cells/ml. For the identification of T cell subsets samples were immunostained for 30 min at 4°C with combinations of the following cell-surface marker antibodies: anti-human CD3 PE cy7 (clone: UCHT1; Thermo Fischer, UK); anti-human CD4 Violet (clone: RPA-T4; Thermo Fischer, UK); anti-human CD8 PE (clone:UCHT4; Immunotools, Germany); anti-human CCR7 FITC (clone:150503; R and D Systems, UK); anti-human CD45RA APC (clone: HI100; Biolegend, UK), anti-human CD28 APC (clone:CD28.2; BD Biosciences, UK) and anti-human CD57 FITC (clone:HCD57; Thermo Fischer, UK). A combination of anti-human CD19 PE (clone: HIB19; Thermo Fischer, UK), anti-human CD27 Violet (clone: O323; Thermo Fischer, UK), anti-human IgD FITC (clone: 1A6-2; Thermo Fischer, UK), anti-human CD24 FITC (clone:SN3; Thermo Fischer,UK) and anti-human CD38 PEcy7 (clone: HIT2; Thermo Fischer,UK) were used to identify B cell subsets. A viability dye eflour 780 (Thermo Fischer, UK) was used to gate out dead cells during flow cytometric analysis. Post-staining, cells were washed in PBS twice and were analysed using a Miltenyi MACS Quant flow cytometer (Miltenyi Biotech, UK). Data analysis was performed using FlowJo software.

T cells were defined as CD3^+ve^ cells and 10,000 cells were gated and divided into CD4^+ve^ and CD8^+ve^, which were further divided into four subsets based on CD45RA and CCR7 expression and denoted as naive (CD45RA^+ve^ CCR7^+ve)^, central memory (CD45RA^−ve^ CCR7^+ve^), effector memory (CD45RA^−ve^ CCR7^−ve^) and terminally differentiated effector memory re-expressing RA, EMRA (CD45RA^+ve^ CCR7^−ve^) (gating strategy; Fig. 1A). CD28^−ve^ CD57^+ve^ CD3^+ve^ cells were denoted as senescent T cells. CD19^+ve^ cells were defined as B cells and 5,000 cells were gated and divided into naïve (CD27^−ve^), memory (CD27^+ve^), plasmablasts (CD38^+ve^ CD24^−ve^) and regulatory B cells (CD24^hi^ CD38^hi^) (gating strategy Fig. 5A). The absolute numbers of immune cells were calculated in conjunction with lymphocyte counts for severe and moderate infection patients.

### Regulatory T cells and Th17 cells

Thawed PBMCs (1 × 10^6^ cells/ml) resuspended in 50 µl of PBS were stained with anti-human CD3 PEcy7, and anti-human CD4 Violet for 30 min at 4 °C. Post incubation, the cells were washed in PBS twice and fixed with Foxp3 Fix Perm solution (Thermo Fischer) for 30 min at room temperature, followed by a wash and staining with anti-human Foxp3 PE (clone: PCH101; Thermo Fischer) and anti-human RORγt APC (clone: 2A2; Thermo Fischer) in diluted permeabilisation buffer (Thermo Fischer) for 30 min at 4 °C. Regulatory T cells were defined as CD3^+ve^ CD4^+ve^ Foxp3^+ve^ cells (gating strategy Fig. 4B) and Th17 cells as CD3^+ve^ CD4^+ve^ RORγt^+ve^ cells (gating strategy Fig. 4D).

### IMM-AGE score calculation

Eight immune cell types (total T cells, naive CD4 T cells, effector memory CD4 and CD8 T cells, EMRA CD8 T cells, CD28^−ve^ CD8 T cells, CD57^+ve^ CD8 T cells and regulatory T cells) were selected to generate the IMM-AGE metric, this is a modified profile from the original scoring that had 20 components [24] that we have reported recently [35]. Only samples that did not have missing values that are required for the IMM-AGE flow calculation were used.

### RNA isolation and Nanostring nCounter gene expression analysis

Total RNA was isolated from 2 × 10^6^ PBMCs from healthy controls and severe COVID-19 convalescent patients using the RNeasy Mini isolation kit (Qiagen, Germany). RNA concentrations and quality were measured using the Agilent 2100 BioAnalyzer. Gene expression analysis was performed using the Pan-Cancer Immune Profiling Panel from NanoString technologies (NanoString, USA). The panel contains probes for 730 immune-related genes and 40 housekeeping genes, representing 24 different immune cell types and common checkpoint inhibitors, covering both adaptive and innate immune responses. For each sample, 80 ng of total RNA, with a maximum of 7 μL (> 28.6 ng/μL), was used. Hybridisation was performed at 65˚C for 17 h using a SimpliAmp Thermal Cycler (Applied Biosystems, UK). The nCounter Flex system (NanoString, USA) was used for sample preparation. Raw gene counts were normalised using the most stable housekeeping genes from the panel. The background threshold was determined as the average count of the negative controls + 2 standard deviations. Differential expression of genes between PBMC from the two cohorts was tested with Mann–Whitney U tests and Benjamin-Hochberg procedures were used to correct for multiple testing. Differentially expressed (DE) genes were further analysed and all pathway analysis was performed within RStudio. Pathway enrichment analysis was performed on a subset of genes differentially expressed between the healthy volunteer and severe COVID-19 groups. This was done using ReactomePA [72]. The BH false discovery method was used and a *p*-value cut-off of < 0.05 was set as significant. Entrez gene IDs were obtained using the org.Hs.eg.db annotation package (http://bioconductor.statistik.tu-dortmund.de/packages/3.10/data/annotation/html/org.Hs.eg.db.html).

### Statistical analysis

All statistical analyses were performed using GraphPad Prism software version 9.2.0. Data distribution was examined using the Kolmogorov–Smirnov normality test. For normally distributed data, a student t-test, or a one-way ANOVA with Bonferroni multiple comparison post hoc tests were performed where appropriate. Relationships between categorial variables were assessed using a Chi-squared test. Multiple linear regression was performed to test for associations between immune parameters and other variables. The probability value (*p*-value) of the statistical significance of the test was used as *p* ≤ 0.05.

### Supplementary Information

**Additional file 1: Supplementary Figure 1.** The long-term impact of COVID-19 on CD8 T cell subset distribution. Comparison of circulating numbers of: (A) CD8 T cells; (B) Naïve; (C) Memory; (D) Central memory; (E) Effector memory; (F) EMRA CD8 T cells between healthy age and sex matched controls (*n* = 39), moderate (*n* = 14) and severe (*n* = 46) COVID-19 convalescent patients. If not indicated, *p* value is not significant.** Supplementary Figure 2.** The long-term impact of COVID-19 on CD4 T cell subset distribution. Comparison of the systemic numbers of: (A) CD4 T cells; (B) Naïve CD4 T cells; (C) Memory CD4 T cells; (D) Central memory CD4 T cells; (E) Effector memory CD4 T cells; (F) EMRA CD4 T cells in healthy age and sex-matched controls (*n* = 39), moderate (n = 14) and severe (*n* = 46) COVID-19 survivors 3-5 months post-infection. Statistical analysis by two-sided Mann–Whitney non-parametric test. If not indicated, p-value is not significant.** Supplementary Figure 3.** CD4 T cell senescence and exhaustion post-COVID-19. Comparison of: (A) absolute numbers of CD28^-ve^CD57^+ve^ CD8 T cells in healthy age and sex matched controls (*n* = 39), moderate (*n* = 14) and severe (*n* = 46) COVID-19 convalescent patients; (B) percentage of CD28^-ve^CD57^+ve^ CD4 T cells in (*n* = 59) and mild (*n* = 15), moderate (*n* = 29) and severe (*n* = 55) COVID-19 survivors 3-5 months post-infection; (C) absolute numbers of CD28^-ve^CD57^+ve^ CD4 T cells in healthy age and sex matched controls (*n* = 39), moderate (*n* = 14) and severe (*n* = 46) COVID-19 convalescent patients; (D) absolute numbers of KLRG1^+ve^ CD8 T cells in healthy age and sex matched controls (*n* = 39), moderate (*n* = 14) and severe (*n* = 46) COVID-19 convalescent patients; (E) percentage of KLRG1^+ve^ CD4 T cells in (*n* = 59) and mild (*n* = 15), moderate (*n* = 29) and severe (*n* = 55) COVID-19 survivors 3-5 months post-infection; (F) absolute numbers of KLRG1^+ve^ CD4 T cells in healthy age and sex matched controls (*n* = 39), moderate (*n* = 14) and severe (*n* = 46) COVID-19 convalescent patients. (G) percentage and (H) absolute numbers of PD1^+ve^ CD4 T cells in healthy age and sex-matched controls (*n* = 21) and severe (*n* = 18) COVID-19 convalescent patients. Statistical analysis by two-sided Mann–Whitney non-parametric test. If not indicated, p-value is not significant.** Supplementary Figure 4.** B cell subsets post-COVID-19. Absolute numbers of systemic: (A) memory B cells; (B) plasma B cells; (C) regulatory B cells in healthy age and sex-matched controls (*n* = 39), moderate (*n* = 14) and severe (*n* = 46) COVID-19 convalescent patients. Statistical analysis by two-sided Mann–Whitney non-parametric test. If not indicated, p-value is not significant.

**Additional file 2.**

## Data Availability

All data generated or analysed during this study are included in this published article and its supplementary information files.
